# Improved survival after introduction of chemotherapy for malignant pleural mesothelioma in Slovenia: Population-based survey of 444 patients

**DOI:** 10.2478/v10019-012-0032-0

**Published:** 2012-05-30

**Authors:** Viljem Kovac, Matjaz Zwitter, Tina Zagar

**Affiliations:** 1 Department of Radiotherapy; 2 Cancer Registry of Republic of Slovenia, Institute of Oncology, Ljubljana, Slovenia

**Keywords:** malignant pleural mesothelioma, incidence, survival, chemotherapy, gemcitabine in prolonged infusion

## Abstract

**Background:**

Malignant pleural mesothelioma is a rare tumour with increasing frequency throughout the world. Due to long latency after exposure to asbestos, restrictions in the production and use of asbestos have not yet alleviated the burden of mesothelioma. During the last decade, several trials confirmed the benefit of systemic treatment with drugs such as doublets with cisplatina and gemcitabine or pemetrexed for carefully selected patients in good performance status. The purpose of this survey was to assess the impact of systemic treatment for the whole national population of patients with mesothelioma.

**Patients and methods.:**

A retrospective study included all patients in Slovenia with histologically confirmed diagnosis of malignant pleural mesothelioma in the period from 1974 till 2008. Data from the Cancer Registry of Slovenia were supplemented by review of clinical records of the Institute of Oncology in Ljubljana where virtually all non-surgical treatment for mesothelioma was performed. We analysed the incidence, treatment, and survival of patients treated in the era of infrequent chemotherapy (1974–2003, the first period) and after it (2004–2008, the second period).

**Results:**

The survey included 444 patients, of whom 325 and 119 were diagnosed in the first and second period, respectively. Joinpoint regression analysis showed that after 1995 the trend in crude incidence rates increased more rapidly; the annual change was 0.03 per 100,000 per year before 1995 and 0.06 per 100,000 per year after. There was clear male predominance (70%) throughout the period covered by the survey. The proportion of patients above 65 years of age increased from 41.8% to 54.6% for the first and second period, respectively (p = 0.02). With a total of 52 (11.7%) operated patients, surgical treatment was rare and used only for selected patients with early disease and without comorbidity, leading to their relatively long median survival of 13.6 months. Chemotherapy was applied to 56 (17.2%) and to 96 (80.7%) patients during the first and second period, respectively. While a variety of older drugs were used in the first period, the most common regimen in the second period (applied to 91 patients) was doublet of low-dose gemcitabine in prolonged infusion and cisplatin. For the whole population of patients regardless the mode of treatment, median survival was 7.4 and 12.6 months (p-value = 0.037) for the first and second period, respectively.

**Conclusions:**

Increasing incidence, male predominance and increased proportion of older patients confirm that the burden of mesothelioma persists in spite of a 15-years old ban in the production of asbestos. Modern chemotherapy, and in particular treatment with low-dose gemcitabine in prolonged infusion and cisplatin significantly prolonged median survival of patients with malignant pleural mesothelioma in Slovenia.

## Introduction

Malignant pleural mesothelioma (MPM) is a rare and highly aggressive tumour arising from mesothelial surfaces of pleura.^1^,^2^

After recognizing asbestos as the most important factor in the pathogenesis of mesothelioma, the production and use of asbestos were banned in most developed countries. However, due to the long latent period between exposure to asbestos and the development of the disease, the incidence of mesothelioma is expected to increase for at least another decade. Since the risk for development of mesothelioma persists for several decades after professional or environmental exposure, the burden of the disease will shift to the older population.^2^–^6^

In spite of all efforts to find an effective treatment, the prognosis of mesothelioma remains poor and over 90% of patients die from the disease.^7^ For the few patients in good performance status, without significant co-morbidity and with apparently limited disease, magnetic resonance (MR) and positron emission tomography-computerised tomography (PET-CT) are helpful in selecting patients with early stages for surgery and/or multimodality therapy with curative intent.^8^–^12^ However, even most aggressive treatment rarely leads to cure. In several series of early-stage mesothelioma treated with multi-modality approach including surgery, median survival rarely exceeds two years. The optimal selection of the surgical procedure remains to be defined and the standard treatment for early stage of MPM remains unclear.^12^–^15^

At the time of diagnosis, most patients have advanced disease. In this situation, systemic treatment is the only modality with a potential to influence survival. Due to scepticism regarding clinical benefit of chemotherapy for patients with mesothelioma, a randomised clinical study was conducted in England, comparing chemotherapy with navel-bine to best supportive care alone. While the trial did not confirm a statistical significant difference, it did show a clear trend for an improved survival for patients treated with chemotherapy, in comparison to the control arm.^16^

During the past 15 years, dozens of trials of chemotherapy for patients with mesothelioma have been reported. Since all these trials were performed on selected populations of patients and none of them included a control arm without chemotherapy, the real value of chemotherapy remains unknown. This is especially true for patients in poor performance status or with significant co-morbidity who are not eligible for large multi-center clinical trials.^17^

In Slovenia, we had two distinct periods of treatment of mesothelioma. Until 2003, occasional patients with early disease were treated with surgery, some patients received palliative irradiation, and very few patients received any form of systemic treatment. In 2003, we activated a Phase II clinical trial of low-dose gemcitabine in prolonged infusion and cisplatin.^18^ Due to the national policy of referral of all patients with mesothelioma to the Institute of Oncology Ljubljana, virtually all patients with mesothelioma eligible for treatment with platin-based chemotherapy were included in the trial.

So far, we found only one population-based report, which has been published to prove that chemotherapy can improve survival for patients with mesothelioma.^19^ We therefore did the following survey, aiming to evaluate the role of chemotherapy for pleural mesothelioma on a population basis in Slovenia.

## Patients and methods

The survey included all patients with permanent residence in Slovenia with a diagnosis of malignant pleural mesothelioma in the period from 1974 till 2008 and reported to the Cancer Registry of Republic of Slovenia. Data were derived from individual hospital reports to the Cancer Registry. For patients who received any form of specific anticancer treatment, additional data were obtained from the clinical documentation of the Institute of Oncology Ljubljana. Eligible patients had biopsy-proven malignant mesothelioma regardless the histologic subtype. Almost all patients had thoracoscopy or CT-guided biopsy as well as US-guided needle biopsy.^18^,^20^,^21^ Stage of disease was not consistently recorded in the clinical documentation and was therefore omitted from the analysis. Data on surgery and on the type of chemotherapy were recorded. During the period covered by the survey, radiotherapy was exclusively applied for palliation, using a wide spectrum of fractionation schedules. Since palliative radiotherapy does not influence survival, data on radiotherapy were not included in the analysis.

The two periods of treatment were defined as the era of infrequent chemotherapy (1974–2003) and of frequent use of chemotherapy (2004–2008). These were further divided into 5-year periods. December 1, 2011 was the close-out date for data collection.

Overall survival time was calculated from the day of diagnosis to the death from all causes or when censored. Kaplan-Meier method was used for estimation of survival and log-rank test was used to compare survival distributions between samples. A p-value lower than 0.05 was considered statistically significant. The data were analysed using SPSS statistical package (Release 13.0, SPSS Inc., Chicago, IL).

The investigators strictly followed recommendations of the Helsinki Declaration (1964, with later amendments) and of the European Council Convention on Protection of Human Rights in Bio-Medicine (Oviedo 1997).

To assess trend in cancer rates, joinpoint regression analysis^22^ was performed with software Joinpoint Regression Program, Version 3.0.

## Results

### Patients

**FIGURE 1:**
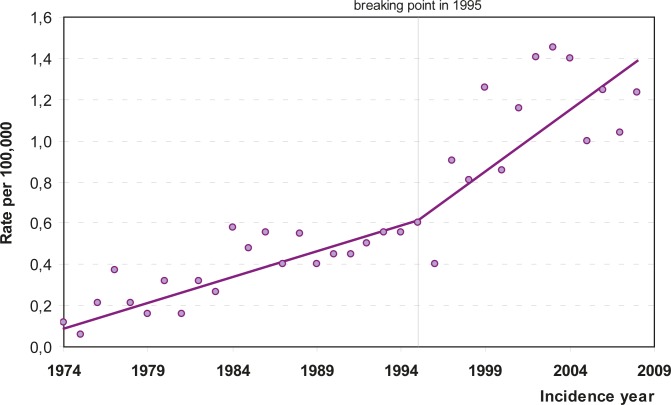
The crude incidence rates of patients with malignant pleural mesothelioma with results of trend analysis, Slovenia 1974–2008.

The survey included 444 patients, of whom 325 had the diagnosis of malignant pleural mesothelioma in the first period (January 1974 – December 2003) and 119 in the second period (January 2004 – December 2008). The incidence increased throughout the analyzed time period (Figure 1, Table 1). Joinpoint regression analysis showed that after 1995, the trend in crude incidence rates increased more rapidly. The annual change in crude rates was 0.03 per 100,000 per year for the 1974–1995 period and 0.06 per 100,000 per year for the 1995–2008 period. Both regression slopes and the difference between the slopes are statistically significant with p-values smaller than 0.05 (Figure 1).

Demographic data are presented in Table 1. Male predominance was obvious (70.7%). Difference in gender distribution in two periods 1974–2003 and 2004–2008 was not statistically significant (Pearson Chi-Square test gives p-value of 0.107). However, the mean age was statistically significant different between the two time periods (t-test gave p-value of 0.021, normal distribution for age was confirmed by Kolmogorov-Smirnov Test). Furthermore, the proportion of patients over 65 years of age at the date of diagnosis increased from 41.8% for the first half of the survey period to 54.6% for the later years.

**FIGURE 2:**
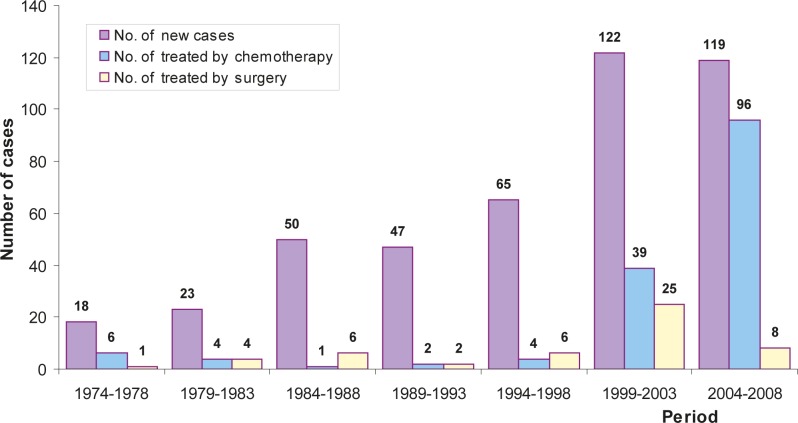
Number of newly diagnosed malignant pleural mesothelioma cases, number of patients treated by chemotherapy and by surgery, Slovenia 1974–2008.

Surgery was rarely used, except for one of the 5-year periods (1999–2003) when the surgical procedures were more frequent (Figure 2).

Fifty-six (17.2%) patients were treated by systemic therapy in the 1974–2003 period and 96 (80.7%) in the years 2004–2008 (Table 2, Figure 2). Two hundreds and sixty-two patients were treated neither by systemic therapy nor by surgery, while 22 patients were treated by both.

The choice of chemotherapy clearly depended on the period. Prior to 2003, only rare patients in unusually good performance status received mono-therapy or doublets with older drugs (cisplatin, doxorubicin, methotrexate, etoposide, or interferon). Exception was 5-year period 1999–2003, when we began with clinical trial and the first 10 patients were treated with a new approach. In the second period, after 2003, 68 patients were enrolled in a Phase II clinical trial and received the doublet of low-dose gemcitabine in prolonged infusion (250 mg/m^2^/6 hours on days 1 and 8) and cisplatin (75 mg/m^2^ on day 2). The similar schedule of treatment was given to 4 patients in another clinical trial Agili. Additional, 19 patients with impaired organ function or in poor performance status who did not meet the eligibility criteria for the trial received a modified treatment schedule. For these poor-risk patients, we usually applied gemcitabine at even a lower dose of 200 mg/m^2^/6 hours and either cisplatin at 60 mg/m^2^ or carboplatin at AUC 5 and omitted gemcitabine on day 8 of a 3-weekly cycle. Finally, 5 patients treated in the last 5 year of survey received other forms of the first line chemotherapy, the doublet of pemetrexed and cisplatin.

### Overall survival

**FIGURE 3:**
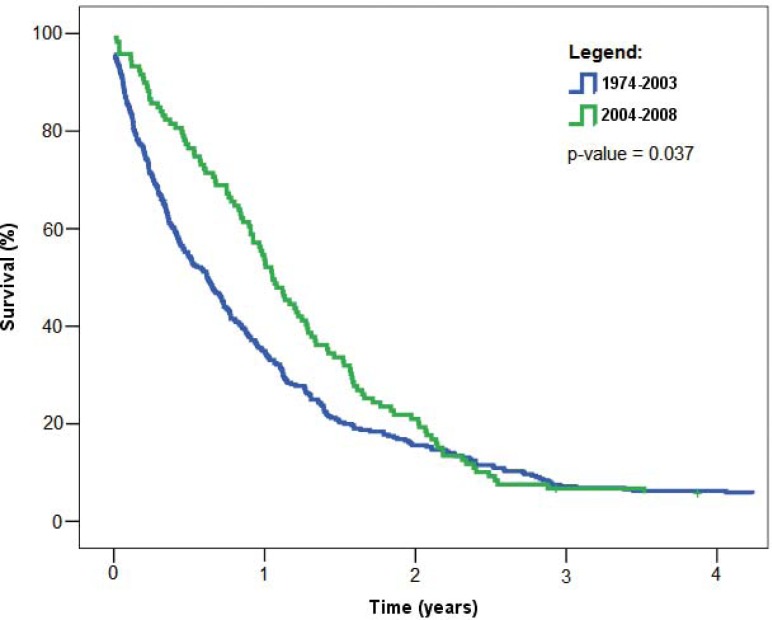
Overall survival of Slovenian patients with malignant pleural mesothelioma by two time periods, 1974–2003 and 2004–2008. P-value refers to log-rank test used to compare survival distributions in the two periods.

Median survival increased from 7.4 months (95% confidence interval [CI] was 5.9–23.8) for the period of 1974–2003 to 12.6 months (95% CI 10.7–14.5) for the period 2004–2008. The difference between the two periods was statistically significant (p = 0.037) (Figure 3).

**FIGURE 4:**
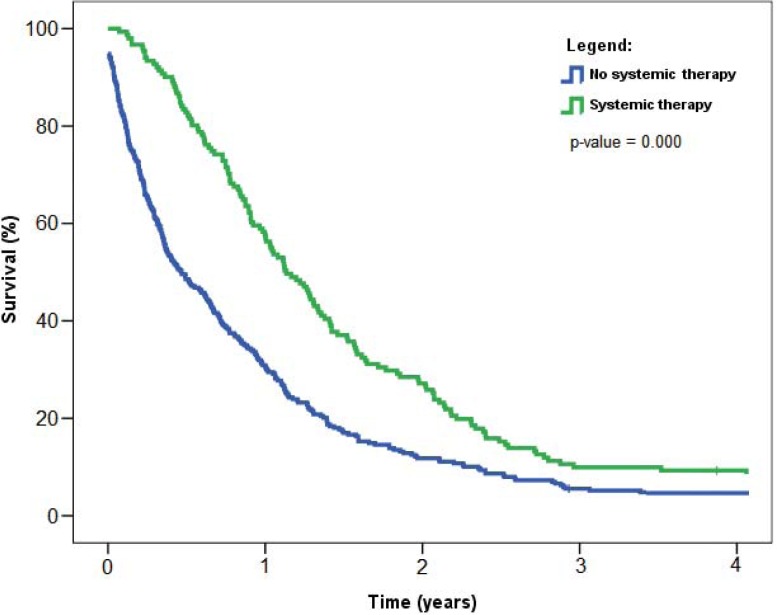
Overall survival of patients with malignant pleural mesothelioma with respect to surgery, Slovenia 1974–2008. P-value refers to log-rank test used to compare survival distributions between the two data samples.

Regarding surgery, the median survival for surgical patients was 13.6 months (95% CI 10.6–16.7), as compared to 8.4 months (95% CI 7.0–9.9) for non-surgical patients (p = 0.000; Figure 4).

**FIGURE 5:**
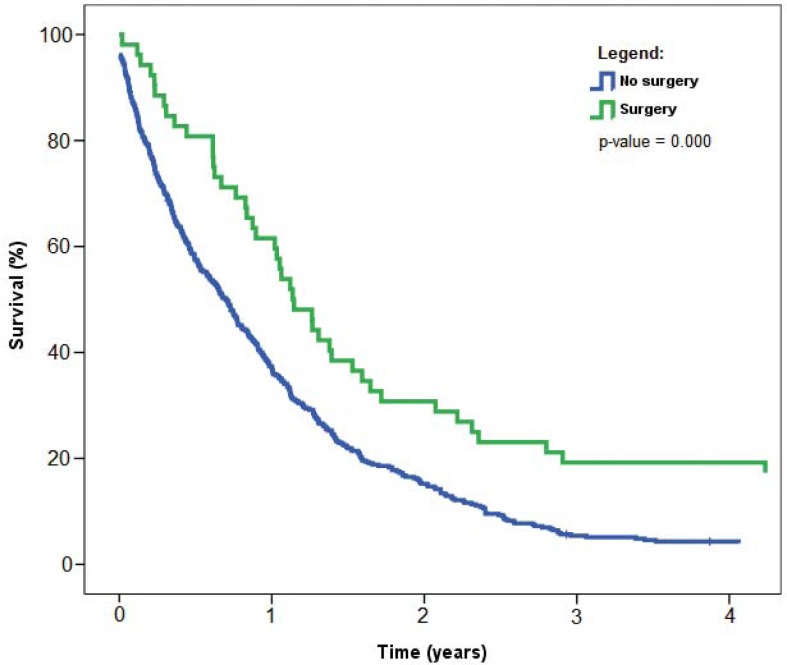
Overall survival of patients with malignant pleural mesothelioma with respect to systemic therapy, Slovenia 1974–2008. P-value refers to log-rank test used to compare survival distributions between the two data samples.

Patients treated by systemic therapy had significantly longer survival than those who did not receive chemotherapy. Median survival times for patients who did receive or did not receive chemotherapy were 14.5 months (95% CI 11.4–15.8) and 5.6 months (95% CI 3.9–7.3), respectively (p = 0.000; Figure 5).

## Discussion

Our survey is the first one, worldwide, to confirmed that systematic introduction of chemotherapy leads to longer survival for the national population of patients with malignant pleural mesothelioma. The whole unselected population as the basis of our survey confirms the validity of the data and makes our survey distinct to reports on clinical trials which typically include only patients in good performance status and without significant co-morbidity.

Slovenia has the privilege of an excellent national cancer registry with a long tradition covering more than 60 years. Moreover, the country is compact, national health policy is well defined, migration of the population is relatively limited, and vital national statistics are complete and reliable. These circumstances further support the validity of the data presented in this survey.

In spite of a ban in the production and use of asbestos implemented in 1996, the incidence of mesothelioma in Slovenia is still rising. Joinpoint regression analysis showed that after 1995 the trend in crude incidence rates increased more rapidly (Figure 1). While better diagnostic possibilities in recent years might contribute to the observation of the rising incidence, we nevertheless believe that the data reflect a real persistent and increasing risk for the disease. Furthermore, our data support the concept of a long latency period between exposure to asbestos and development of mesothelioma. In this respect, we see a persistent and markedly increased risk in the local communities at close proximity to the former asbestos factory.^18^ Also notable is an increasing proportion of elderly patients with mesothelioma and clear male predominance in recent cohorts covered by our survey. At 73 years after the beginning of production of asbestos in Slovenia and 15 years after the facility closed its production of asbestos, these observations additionally indicate that the latency period from the exposure to asbestos to the development of disease is really long.

The other putative aetiological factor, Semian virus 40, was not implicated in pathogenesis of malignant pleural mesothelioma in Slovenia.^23^,^24^

So far, all efforts to implement screening and early diagnosis of mesothelioma for the high-risk populations have failed, or are still in the investigative phase.^2^ Our survey proves that very few patients are diagnosed at an early stage when multi-modality treatment with a curative intent is a realistic option. We believe that carefully selected patients do benefit from surgery; indeed, patients treated with surgery had significantly better survival than those who were not operated (p-value = 0.000) (Figure 4). In the interpretation of these data, one should consider that the surgical patients are usually those with good prognostic factors: good performance status, low comorbidity, low stage of disease, low weight loss and epitheloid subtype of mesothelioma.^18^,^20^,^25^,^26^ A bias in the selection for surgery precludes any comparison to other patients.^18^,^20^

Our survey revealed a statistically superior survival for patients treated after 2004 when we introduced chemotherapy as a standard treatment modality for mesothelioma. Regarding this finding, two possible factors leading to a bias should be discussed. The first one is earlier diagnosis in recent cohorts of patients. This seems unlikely, since there was no program for early diagnostics of mesothelioma and since the number of patients with early operable stages remained constantly low. The second possible bias is improved supportive care in recent years. While this possibility cannot be entirely rejected^27^, we believe that better supportive care alone cannot be responsible for a prolongation of the median survival for more than 5 months. Hence, it seems reasonable to link improved survival to the new treatment policy and to introduction of chemotherapy.

After the trial conducted in England and discussed in the introduction^16^ and after our survey^18^, the question of benefit of chemotherapy for most patients with mesothelioma appears to be solved. However, the choice of a particular chemotherapeutic schedule is a distinct question. The three parameters determining the choice are efficacy; side effects, quality of life and convenience for the patients; and costs. We will first discuss the published experience with other scheduled of chemotherapy and later return to low-dose gemcitabine in prolonged infusion and cisplatin as our preferred combination during the last five years of our survey.

In 2003, Vogelzang published experience from a landmark trial which compared pemetrexed and cisplatin against monotherapy with cisplatin and demonstrated a statistically significant advantage for the doublet.^28^ On the basis of this trial, pemetrexed was the first drug to be specifically registered for the treatment of mesothelioma, leading to its wide acceptance as the standard treatment. A critical look reveals that the superiority of pemetrexed may be attributable to suboptimal control arm: cisplatin alone is was never the standard treatment for mesothelioma, and certainly not at the turn of the century when several other drugs and their combinations were available. In that period, the doublet of gemcitabine and cisplatin or carboplatin was the most widely used systemic treatment for mesothelioma patients.^29^ Pooled data of 7 studies lead to an estimated median survival of 11.7 months, which is comparable with median survival of 12.1 months in pemetrexed study.^18^,^28^ A large spectrum of other combinations from various Phase II clinical trials reported results which are at least comparable to the doublet of pemetrexed and cisplatin, and superior to cisplatin alone (Table 3).

Superior survival of the national pool of patients with mesothelioma during the last five years of our survey should be attributable to low-dose gemcitabine in prolonged infusion and cisplatin as our preferred combination. On the basis of a favourable experience in several trials for non-small cell lung cancer^50^–^52^, we decided to use this combination also for patients with mesothelioma and included 78 patients in a Phase II clinical trial (10 patients in the 5-year period 1999–2003 and 68 patients in the 5-year period 2004–2008).^18^ In the last 5-year period, additional 19 patients in poor performance status or with organ dysfunction who were not eligible for the aforementioned trials received a less intensive modification of the same schedule. After completing a Phase II trial for mesothelioma, our research continues with an on-going randomised Phase II trial which compares this combination to the doublet of pemetrexed and cisplatin (**A**limta vs. **G**emcitabin **I**n **L**ong **I**nfusion – AGILI trial).^53^

During the last 5 years of the survey, treatment with low-dose gemcitabine in long infusion and cisplatin was applied to a total of 91 patients. This figure represents 94.8% of the total number of patients who received any form of chemotherapy and 76.5% of the total number of patients with mesothelioma during this period (91 out of 119). Future clinical research on mesothelioma should address several important questions. One of them is to compare different chemotherapy schedules for their efficacy and tolerability, a question already addressed in our on-going AGILI trial.^53^ The second one is the question of maintenance treatment. This concept got wide acceptance in the treatment of advanced non-small cell lung cancer.^54^,^55^ Regarding mesothelioma, several trials (including our Phase II trial of low-dose gemcitabine in prolonged infusion and cisplatin) showed that patients who responded to first-line treatment have fair chances to benefit either from re-induction of the same treatment, or from a different combination of drugs.^18^,^56^,^57^ Finally, research should focus on genetic polymorphisms which influence DNA damage^58^, leading to individualised systemic treatment. A key issue in the development of individualized therapy is identification of biomarkers to predict chemotherapeutics’ efficacy and toxicity.^59^,^60^ Thus, our research on patients with mesothelioma confirmed that the nucleotide excision repair (NER) pathway polymorphisms influence platinum-treatment efficacy and toxicity^26^ and that ribonucleotide reductase subunit 1 (RRM1) polymorphisms as well as haplotypes are associated with gemcitabine treatment efficacy and toxicity.^60^

In conclusion, our survey showed superior survival of patients with mesothelioma during the last five years when a new national policy was implemented and virtually all eligible patients received chemotherapy. Our success should be attributable to our preferred schedule of low-dose gemcitabine in prolonged infusion which proved to be effective, with acceptable toxicity also for patients in poor performance status, and linked to reasonable costs.

## Figures and Tables

**TABLE 1:** Demographic data for malignant pleural mesothelioma, Slovenia 1974–2008

	**1974–2003**	**2004–2008**	**Total**
**Males**: number (%)	223 (68.6%)	91 (76.5%)	314 (70.7%)
**Females**: number (%)	102 (31.4%)	28 (23.5%)	130 (29.3%)
**Age**: range (mean)	22–89 (61.7)	33–87 (64.4)^*^	22–89 (62.4)
**Age above 65**: number (%)	136 (41.8%)	65 (54.6%)	201 (45.3%)
**Total**	**325**	**119**	**444**

* p = 0.021

**TABLE 2:** Number of newly diagnosed malignant pleural mesothelioma cases, number of patients treated by chemotherapy and by surgery, Slovenia 1974–2008

	**No. of new cases**	**No. of treated by systemic therapy (%)**	**No. of treated by surgery (%)**
**1974–1978**	18	6 (33.3%)	1 (5.6%)
**1979–1983**	23	4 (17.4%)	4 (17.4%)
**1984–1988**	50	1 (2.0%)	6 (12.0%)
**1989–1993**	47	2 (4.3%)	2 (4.3%)
**1994–1998**	65	4 (6.2%)	6 (9.2%)
**1999–2003**	122	39 (32.0%)	25 (20.5%)
**2004–2008**	119	96 (80.7%)	8 (6.7%)
**Total**	**444**	**152 (34.2%)**	**52 (11.7%)**

**TABLE 3:** Effectiveness of different chemotherapy schemes in the treatment of the patients with malignant pleural mesothelioma

**Trial**	**Phase of study**	**Drugs**	**N**	**RR, %**	**MOS, months**	**MPFS, months**	**1-year survival, %**
Byrne MJ *et al.*^30^, 1999	Phase II	Gemcitabine + cisplatin	21	48	10.0	NA	NA
Aversa SM *et* Favaretto AG^31^, 1999	Phase II	Gemcitabine + carboplatin	20	20	NA	4–21	NA
van Haarst JMW *et al.*^32^, 2002	Phase II	Gemcitabine + cisplatin	32	16	9.6	6.0	36
Nowak AK *et al.*^33^, 2002	Phase II	Gemcitabine + cisplatin	52	17	11.2	6.4	NA
Mikulski SM *et al*.^34^, 2002	Phase II	Ranpirnase	105	5	8.3	3.4	34
Vogelzang NJ *et al.*^28^, 2003	Phase III	Pemetrexed + cisplatin	226	41	12.1	5.7	50
		cisplatin	222	17	9.3	3.9	38
Favaretto AG *et al*.^35^, 2003	Phase II	Gemcitabine + carboplatin	50	26	14.7	8.9	53
Andreopoulou E *et al.*^36^, 2004	Phase II	Mitomycin C + vinblastine + cisplatin	150	15	7.0	NA	31
van Meerbeeck JP *et al.*^37^, 2005	Phase III	Raltitrexed + cisplatin	126	24	11.4	5.3	46
		cisplatin	124	14	8.8	4.0	40
Castagneto B *et al.*^38^, 2005	Phase II	Gemcitabine + cisplatin	35	26	13.0	8.0	NA
Berghmans T *et al*.^39^, 2005	Phase II	Epirubicin + cisplatin	69	19	13.3	NA	50
Jänne PA *et al*.^40^, 2005	Phase II	Pemetreksed+gemcitabine	108	17	10.1	7.4	46
Ceresoli GL *et al.*^41^, 2006	Phase II	Pemetrexed + carboplatin	102	19	12.7	6.5	52
Obasaju CK *et al.*^42^, 2007	Phase IV (EAP)	Pemetrexed + cisplatin	728	21	10.8	NA	45
Santoro A *et al.*^43^, 2007	Phase IV (EAP)	Pemetrexed + cisplatin or carboplatin	861	22	NA	NA	64
Castagneto B *et al.*^44^, 2008	Phase II	Pemetrexed + carboplatin	76	25	14.0	NA	NA
Kalmadi SR *et al*^45^, 2008	Phase II	Gemcitabine + cisplatin	50	12	10.0	6.0	30
Hillerdal G *et al*^46^, 2008	Phase II	Gemcitabine + carboplatin +	173	32	13.0	8.6	NA
		liposomized doxorubicin					
Sørensen JB *et al*^47^, 2008	Phase II	Vinorelbin + cisplatin	54	30	16.8	7.2	61
Muers M *et al.*^16^, 2008	Phase III	Vinorelbin	136	16	9.5	6.2	34
		Mitomycin + vinblastine + cisplatin	132	10	7.6	5.6	31
Ralli M *et al*.^48^, 2009	Phase II	Docetaxel + gemcitabine	25	28	15.0	7.0	NA
Sørensen JB *et al*^49^, 2011	Phase II	Vinorelbin + carboplatin	47	30	14.6	7.2	55
Kovac V *et al.*^18^, 2012	Phase II	Gemcitabine^*^ + cisplatin	78	50	17.0	8.0	67

N = number of patients included in the trial; RR = response rate; MOS = median overall survival; MPFS = median progression-free survival, NA = not available; EAP = expanded access program

* applied in low dose in 6-hours infusion
